# Ethical clinical trial design and differences in treatment effects

**DOI:** 10.2471/BLT.24.292177

**Published:** 2025-08-25

**Authors:** Roger J Lewis, Kert Viele, Margareth Ndomondo-Sigonda, Samba Sow, Elvis Temfack, Nathalie Strub-Wourgaft

**Affiliations:** a Berry Consultants, LLC, 3345 Bee Caves Rd, Suite 201, Austin, Texas 78746, United States of America.; b Faculty of Health Sciences, University of Witwatersrand, Johannesburg, South Africa.; c Centre pour les Vaccins en Développement, Bamako, Mali.; d Center for Science and Innovation, Africa Centers for Disease Control and Prevention, Addis Ababa, Ethiopia.; e PANTHER (PANdemic preparedness platform for Health and Emerging infections Response), Paris, France.

## Abstract

Many global clinical trials primarily estimate a single overall treatment effect. However, when treatment effects are likely to differ between populations, for example due to differences in the disease, population characteristics or health-care systems, this approach can lead to misleading conclusions and raise ethical concerns. Justice is compromised when research conducted in low-resourced countries benefits primarily or exclusively populations of wealthier countries. A clinical trial design and analysis that focuses on estimating a single treatment effect, assumed to apply to all participating populations, goes against the ethical principle of justice and the positions of the World Health Assembly. To address this issue, we suggest a methodological strategy based on hierarchical modelling. This approach enables researchers to estimate treatment effects that are valid for each participating population, while potentially retaining efficiency comparable to traditional pooled analysis, as we demonstrate in an example. When substantial between-population differences exist, it produces valid, region-specific results. Strategies such as this one, if adopted into the standards for global trials, would allow regulators, funders and other stakeholders to ensure that trials are designed to help preserve justice for all participant populations.

## Résumé

De nombreux essais cliniques menés à l’échelle internationale évaluent principalement un seul effet global d’un traitement. Cependant, lorsque les effets de ce traitement sont susceptibles de varier d’une population à l’autre, par exemple en raison de différences dans l’affection étudiée, les caractéristiques de la population ou les systèmes de santé, cette approche est susceptible de produire des conclusions trompeuses et de soulever des questions éthiques. La justice est compromise lorsque les recherches menées dans les pays à faibles ressources profitent principalement ou exclusivement aux populations des pays plus riches. Le design et l’analyse d’un essai clinique qui se concentre sur l’estimation d’un seul effet thérapeutique, censé s’appliquer à toutes les populations participantes, vont à l’encontre du principe éthique de justice et des positions de l’Assemblée mondiale de la Santé. Pour remédier à ce problème, nous proposons une stratégie méthodologique axée sur la modélisation hiérarchique. Cette approche permet aux chercheurs d’estimer les effets du traitement qui sont valables pour chaque population participante, tout en conservant potentiellement une efficacité comparable à celle de l’analyse groupée traditionnelle, comme nous le démontrons dans un exemple. En présence de différences considérables entre les populations, elle produit des résultats valables et spécifiques à chaque région. Des stratégies telles que celle-ci, si elles étaient adoptées dans les normes applicables aux essais cliniques mondiaux, permettraient aux régulateurs, aux bailleurs de fonds et aux autres parties prenantes de s’assurer que le design des essais préserve l’équité pour toutes les populations participantes.

## Resumen

Muchos ensayos clínicos internacionales estiman principalmente un único efecto global del tratamiento. Sin embargo, cuando es probable que los efectos del tratamiento difieran entre poblaciones, por ejemplo, debido a variaciones en la enfermedad, las características de la población o los sistemas de salud, este enfoque puede conducir a conclusiones engañosas y suscitar preocupaciones éticas. La justicia se ve comprometida cuando la investigación realizada en países con recursos limitados beneficia principalmente o de manera exclusiva a poblaciones de países más ricos. El diseño y el análisis de un ensayo clínico centrado en estimar un único efecto del tratamiento, asumido como aplicable a todas las poblaciones participantes, contraviene el principio ético de justicia y las posiciones de la Asamblea Mundial de la Salud. Para abordar este problema, se plantea una estrategia metodológica basada en el modelado jerárquico. Este enfoque permite a los investigadores estimar efectos del tratamiento válidos para cada población participante, al tiempo que se conserva una eficiencia comparable con la del análisis combinado tradicional, como se ilustra en un ejemplo. Cuando existen diferencias sustanciales entre poblaciones, se obtienen resultados válidos y específicos para cada región. La adopción de estrategias como esta en los estándares de los ensayos clínicos internacionales permitiría a los organismos reguladores, financiadores y demás partes interesadas garantizar que los ensayos se diseñen de manera que contribuyan a preservar la justicia para todas las poblaciones participantes.

## ملخص

تقوم العديد من التجارب الإكلينيكية العالمية بشكل أساسي بتقدير تأثير علاجي شامل وحيد. ومع ذلك، عندما يكون من المحتمل اختلاف آثار العلاج بين المجموعات السكانية، على سبيل المثال بسبب اختلافات في المرض، أو خصائص السكان، أو أنظمة الرعاية الصحية، فإن هذا الأسلوب قد يؤدي إلى استنتاجات مضللة، ويثير مخاوف أخلاقية. يتم الإخلال بالعدالة عندما تستفيد الأبحاث التي تُجرى في الدول ذات الدخل المنخفض، من الموارد بشكل أساسي أو حصري لصالح سكان الدول الأغنى. إن تصميم وتحليل التجارب الإكلينيكية التي تركز على تقدير تأثير علاجي واحد، والتي تفترض أنه ينطبق على جميع السكان المشاركين، يتعارض مع المبدأ الأخلاقي للعدالة ومواقف جمعية الصحة العالمية. ولمعالجة هذه المشكلة، فإننا نقترح استراتيجية منهجية قائمة على وضع النماذج التسلسلية. يتيح هذا الأسلوب للباحثين تقدير آثار العلاج المناسبة لكل مجموعة سكانية مشاركة، مع الحفاظ على كفاءة تُضاهي التحليل التجميعي التقليدي، كما هو موضح في أحد الأمثلة. وعندما توجد اختلافات جوهرية بين السكان، فإنها تقدم نتائج صالحة خاصة بكل منطقة. إن مثل هذه الاستراتيجية، إذا تم اعتمادها ضمن المعايير القياسية للتجارب العالمية، من شأنها أن تسمح للجهات التنظيمية، وجهات تحصيل التمويل، وأصحاب المصلحة الآخرين، بضمان أن التجارب مصممة للمساعدة في الحفاظ على العدالة لجميع السكان المشاركين.

## 摘要

大部分全球临床试验主要是为了评估单一整体疗效。但是，鉴于群体之间的疗效可能会存在差异（例如由于疾病类型、群体特性或医疗体系的不同），采用该方法可能会导致得出误导性结论并引发伦理问题。当在资源匮乏国家开展的研究的受益对象主要或仅仅是较富裕国家的国民时，此类研究已失去公正性。侧重于评估单一疗效的临床试验设计与分析（假设适用于所有参与群体）违背了世界卫生大会所倡导伦理原则中的公正原则及其主张的立场。为解决这一问题，我们建议基于分层模型采用方法论策略。研究人员可采用该方法评估是否对所有参与群体都具有显著疗效，同时正如我们在示例中演示的，该方法的效率可能与传统汇集分析不相上下。当不同群体之间存在显著差异时，可能会产生具有区域特异性的有效结果。如果此类策略被纳入全球试验标准中，则监管机构、出资方以及其他利益攸关方均能确保试验设计有助于维护所有参与群体之间的公正性。

## Резюме

Многие клинические исследования, проводимые во всемирном масштабе, в первую очередь оценивают один общий эффект лечения. Однако, если имеется вероятность того, что эффекты лечения будут различаться для разных популяций (например, из-за различия в заболевании, разнообразия популяционных характеристик или отличий в системах здравоохранения), такой подход может привести к неправильным выводам и вызывать этические конфликты. Принцип справедливости нарушается, когда исследования, проводимые в странах с низким уровнем доходов, в итоге приносят пользу преимущественно или исключительно населению более богатых стран. Дизайн клинических исследований и анализ данных, в котором принимается во внимание только один эффект от лечения в предположении, что он применим ко всем участвующим популяциям, нарушает этический принцип справедливости и не соответствует позиции Всемирной ассамблеи здравоохранения. Для того чтобы решить эту проблему, авторы предлагают методологическую стратегию, основанную на иерархическом моделировании. Такой подход позволяет исследователям оценивать эффекты лечения, которые будут действительными для каждой из участвующих популяций, но потенциально может при этом сохранять эффективность, сравнимую с традиционным анализом по обобщенным данным, как показано на примере. При наличии существенных популяционных различий такой метод дает возможность получить валидные результаты, отражающие специфику конкретных регионов. Если такие стратегии, как эта, будут включены в стандарты для всемирных исследований, это даст возможность регуляторным органам, финансовым спонсорам и другим участникам процесса гарантировать такой дизайн исследований, который будет справедливым для всех участвующих популяций.

## Introduction

The benefit of medical interventions may vary across settings, for example due to differences in patient populations, disease characteristics and health-care systems. This heterogeneity of treatment effect complicates the design and interpretation of global clinical trials. Quantifying the treatment effect or achieving adequate statistical power separately in each setting, rather than just for the trial as a whole, requires a substantially larger number of participants.^1^^,^^2^ For reasons of practicality, trials are often designed to estimate a single, overall trial treatment effect, with a plan to conduct exploratory and often underpowered analyses of benefit across different settings. These approaches are associated with increased risk of both false-positive and false-negative errors, making interpretation of results challenging.^1^^,^^2^ This tension between the goals of quantifying setting-specific treatment effects and practical considerations has both ethical and statistical dimensions.

For example, the global mpox outbreak that began in 2022 has been characterized by substantial differences in viral clades, modes of transmission and populations across regions, each of which may affect the benefit of antiviral therapies.^3^ In response to the outbreak, several clinical trials were launched to evaluate the effect of tecovirimat treatment, with the expected generation of data on the benefit of tecovirimat in multiple study settings. This situation prompted the consideration of how best to analyse and interpret the data collected, in a manner that would best inform treatment decisions specific to each region.

### Justice in medical research

The concept of justice in medical research implies that those who bear the risks and burdens of participation in research should benefit from the resulting knowledge.^4^ Similarly, those who benefit from the resulting knowledge should bear the risks and burdens of participation in the research.^4^ Justice is compromised when research is conducted in populations of low- and middle-income countries and the results of that research primarily or exclusively benefit the populations of wealthier nations. This injustice may occur when the therapies identified by such research have limited availability where the research was conducted, for example due to cost, limitations in the health system or other constraints.^5^

In May 2022, the World Health Assembly adopted a resolution calling for greater equity in the conduct of clinical trials.^6^ The resolution acknowledges the key role of global, randomized, multicountry clinical trials in efficiently collecting information on treatment efficacy and safety relevant to each region, as well as asserting the importance of including all major population groups the intervention is intended to benefit, with particular focus on under-represented populations.^6^

However, even when a therapy is investigated and intended to be available globally, justice may still be compromised. Justice also implies that clinical trials be designed to provide a valid answer to the relevant research questions in each local context, because not doing so limits the value of the research to one or more of the participating populations.^5^

### Heterogeneity of treatment effect

Geographic differences or heterogeneity in the effects of an investigational treatment may arise from multiple causes, including differences in the disease (viral subtypes), patients (underlying health status, concomitant treatments, genetics and/or social determinants of health) and the health-care system (time to access to care and/or hospital resources). Therefore, it is difficult to predict when treatment effects from geographically distinct settings will be quantitatively indistinguishable, only qualitatively similar or even qualitatively disparate. Our ability to precisely quantify heterogeneity of treatment effect is often limited, for example due to small sample sizes within populations, or because of primary analysis strategies that assume a single common treatment effect across populations.

### Design and analysis strategy

While it is vital that research intended to benefit patients in multiple regions includes meaningful numbers of participants from each region, geographically inclusive research comes with inferential challenges. A common strategy in analysing randomized clinical trials is to use statistical models that account for differences between regions or clinical study sites on overall prognosis, while still pooling information across sites or regions to estimate a single, overall average treatment effect.^7^ Subsequent analyses of subgroups may be conducted to provide descriptive summaries of treatment effects in different subgroups, but these are considered exploratory (Table 1).

**Table 1:** Characteristics of three approaches to addressing regional differences in treatment effect of clinical trials

Considerations	Strategy for assessing heterogeneity of treatment effect by region		
	Pool data for primary analysis, then conduct separate analyses for each region	Hierarchical model to integrate information across regions	Conduct separate primary analyses for each region
Underlying assumptions	Primary analysis assumes a single common treatment effect exists for all regions	Assumes treatment effects in each region are themselves drawn from a common distribution of treatment effects	Assumes that treatment effects in different regions are unrelated
Precision of estimate of treatment effect(s)	Highest precision around the estimated treatment effect; highest power	Precision and power depend on the observed similarity of treatment effects across regions	Low precision around the estimated treatment effect; lowest power. Data from other regions are ignored
Validity of estimated treatment effect within each region	Treatment effect may or may not be valid for any region or population if there is heterogeneity of the treatment effect	Lowest average error in estimated treatment effect across populations; yields valid population-specific estimates of treatment effect^8^^,^^9^	Variability of the treatment effect across regions is likely to be overestimated, potentially falsely suggesting differences in treatment response
Additional considerations	Interpretation is unclear if secondary, separate analyses suggest substantial variability in treatment effects across regions	Treatment effect estimates for populations with small sample sizes may be based mostly on data from other populations, if there is general consistency of treatment effect across populations	Interpretation may be unclear if the sample size in some regions is insufficient, as differences in apparent treatment effect may be real or due to random variability

The typical subgroup analysis starts with a formal pooled analysis, followed by descriptive subgroup analyses in which each region is evaluated in isolation. This approach has important limitations. First, there must be no heterogeneity in the treatment effect for the primary analysis to be valid for all groups of study participants. If heterogeneity exists, the estimate of the overall average treatment effect may not be valid for any identifiable group in the population studied. Second, the independent descriptive analyses of the treatment benefit in each region systematically overestimate the true variability in the treatment effect, since they fail to consider the normal variability expected when obtaining multiple estimates of the intervention’s treatment effect.^8^^,^^9^ This overestimation of variability in treatment benefit could lead to either the withholding of treatments that are truly effective, or the administration of treatments that are ineffective in specific subgroups or regions, based on an apparent local treatment effect that falsely appears different from the others.

An alternative approach for the primary analysis would be to independently analyse the data from each geographical region. This approach may fail to discover treatment effects when present (due to low statistical power in the individual regions). Furthermore, when there is consistency of the observed treatment effect across regions, this approach also fails to leverage that consistency to increase statistical power (Table 1). Conducting individual, standalone studies sufficiently powered for each region is often impossible.^1^^,^^2^^,^^5^^,^^6^^,^^10^

Instead, when analysing information from multiple regions, we should use prespecified statistical methods that recognize and leverage commonality of treatment effects when it is present, while allowing for regional differences when necessary. The goal of this approach is to pool information and maximize statistical power when the treatment effect across regions is consistent, and to consider regions (or subgroups) more independently when doing so is necessary because of differences in treatment benefit. The statistical models must be sufficiently flexible both to recognize which reality appears more likely, and to produce valid statistical estimates tailored to each region.

## Hierarchical statistical models

A hierarchical model can bridge the gap between the two undesirable extremes of either pooling data across settings to generate a single estimate of benefit or analysing each setting’s data in isolation.^8^^,^^11^ The structure of the model allows the population in each setting to be associated with its own treatment effect but, rather than assuming the region-specific treatment effects are unrelated to each other, the model assumes that the setting-specific treatment effects are all representative of a range of treatment effects that would be seen across a large number of hypothetical, affected populations. The model uses the observed data to gain information on the degree of similarity of treatment effects across populations which, in turn, influences the degree of pooling or assumed similarity of treatment effects across populations or regions.^8^^,^^11^ This approach accommodates both the commonality of the human condition and the tendency of treatments that are effective in one setting to be effective in others, and the fact that differences exist between populations, diseases and health-care systems in different settings.

A hierarchical model can provide treatment effect estimates for each setting under study, with these estimates primarily informed by the data in each specific setting, while adjusting for the broader context provided by data from the other settings. A suitably structured hierarchical model is flexible in how it accounts for similarities and differences between the regions. In situations with similar estimates across settings, the hierarchical model recognizes this similarity and produces estimates closer to the pooled average, with higher precision, reflecting the reinforcement that each setting’s estimate receives from replication in the other settings. In situations with widely varying estimates across settings, the hierarchical model generates estimates that more closely reflect each setting’s individual data, allowing for more independent analysis. For example, in a clinical trial including participants from the African and European regions, if data from African populations suggest a treatment effect quite different from the European Region, then the estimate of benefit in African participants would likely be very similar to an estimate based solely on African data.

As an example, consider estimating the treatment effect most relevant for treatment decisions in the African Region, using data from a global clinical trial involving participants in the African Region, the Region of the Americas and the European Region. The data obtained from the African Region is directly relevant to African treatment decisions, but using the information from the two other regions will potentially improve inferences for the treatment of African patients.

In this example, our endpoint is the time to resolution of a disease, similar to mpox, and we assume 250 participants are enrolled in each region and randomly assigned in equal numbers to the experimental and control groups. The resulting data will be analysed using a Cox proportional hazards model or other time-to-event model, with a hazard ratio (HR) greater than 1.0 indicating treatment benefit with more rapid resolution. The hierarchical model is structured to allow the sharing of information on the HRs across regions. Box 1 presents the mathematical details.

Box 1 **Mathematical details for a hierarchical statistical model to estimate treatment effect in global clinical trials**

Let *H_c_(t)* be the hazard function for the control arm in each region *c*. In this case, *c* represents the African Region, the Region of the Americas or the European Region, as a function of time *t*. *H_c_(t)* may be estimated through any common time-to-event method (for example, Cox proportional hazards model or piecewise exponential model). In our example, we use an exponential model for simplicity, but this approach does not alter the qualitative behaviour of the model in terms of the dynamic borrowing of information across regions. The hazard function model may include adjustments for covariates.

The treatment effect in each region is defined by the hazard ratio *HR_c_*, reflecting the relative rate of resolution in the active treatment group to the control group. The hierarchical model is defined by placing a weak informative normal prior distribution with mean *η* and standard deviation *τ*, *N(η,τ)*, on the logs of the three HRs:

*Log(HR_Africa_), log(HR_Americas_), log(HR_Europe_) ~N(*,*)*

*η ~N(M,S)*

*τ^2^ ~InvGamma(α,β)*

with priors placed, in turn, on the parameters of *N(η, τ)* in a hierarchical manner. *N(M,S)* denotes the normal distribution prior for the centre of the distribution of log-HRs with, for the example here, the mean *M*: 0 and the standard deviation *S*: 10. *InvGamma(α,β*) denotes the inverse gamma distribution with parameters, for the example here, *α*: 0.25 and *β*: 0.0025. Many similar prior structures are possible. For example, the inverse gamma prior on *τ^2^* may be replaced with multiple other distributions.

The parameter *τ* is the most important variable of this model, measuring the across-region variation. When *τ* is small, the three regions have similar HRs. When *τ* is large, the HRs may be very different between regions. The prior on *τ* represents a plausible a priori range, and the behaviour of the model can be tuned by carefully selecting this prior distribution to produce both reasonable results within individual trials and reasonable operating characteristics (for example, power and type 1 error) across many trials.

After collecting data, the Bayesian posterior distribution of all the parameters is computed. The differences between the regions, relative to their inherent sampling variability, inform the estimate of *τ*. Informally, the model looks at the differences between the point estimates across regions relative to the widths of the nonborrowing credible intervals. If the differences between regions is explainable by random noise, *τ* is estimated to be smaller, and the model pulls the estimates closer together while increasing precision. If the differences between the regions are greater than expected from random noise, the model borrows minimally, with each region’s data standing alone.

HR: hazard ratio.

In formulating the hierarchical model, we place a Bayesian prior probability distribution on the variation in the treatment effect across the regions. This step sets the a priori range of the plausible magnitude of heterogeneity of the treatment effect, which is then updated or refined once the data are available. If the data are consistent with similar treatment effects across the three continents, the model will share more of the information across the regions, a process often termed borrowing of information or borrowing strength in the statistical literature,^12^ which achieves a result closer to pooling and assuming a single overall treatment effect. In contrast, if the data are more consistent with true differences in the treatment effect across the regions, the model will share less information, yielding estimates that are closer to those that would be obtained with independent analyses in each region. However, in this case, the model appropriately corrects the exaggerated heterogeneity that would occur if data from each region were analysed independently.

**Fig. 1:**
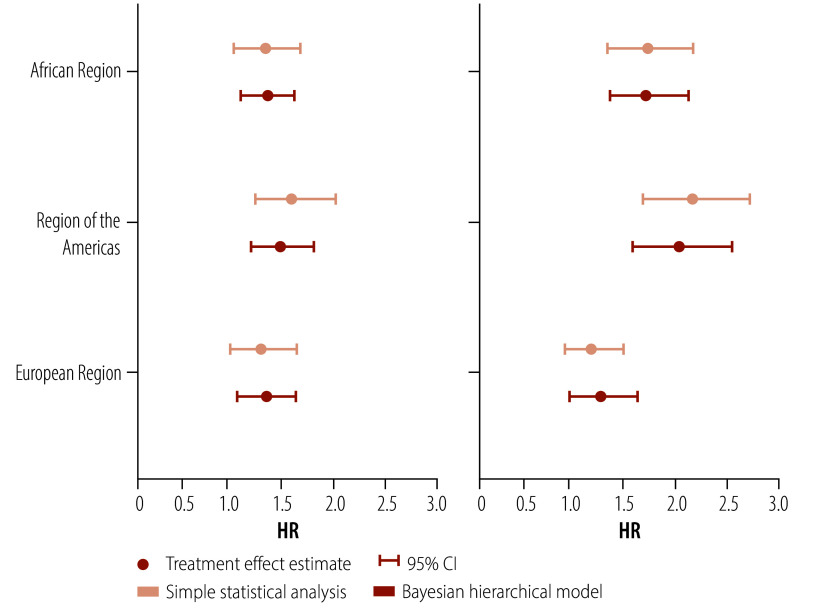
Two examples of clinical trial results of observed treatment effect

CI: credible interval; HR: hazard ratio

Notes: Example 1 shows results that largely agree across regions while example 2 shows greater heterogeneity in the observed treatment effect. In example 1, the similar results lead the model to borrow more information across regions, with the point estimates closer together and the resulting credible intervals considerably smaller than their counterparts without borrowing. In example 2, the large differences between regions result in minimal borrowing, with each region’s estimate being minimally changed by borrowing.

Fig. 1 illustrates the HRs between treatment and control from two example data sets analysed both without borrowing of information and with borrowing of information using the hierarchical model, for each of the three regions. 

Example 1 shows the three regions producing very similar results. Without borrowing, the credible intervals are largely overlapping. In this situation, the model largely attributes differences across regions to random variation and produces point estimates for each region that are very similar. In addition, the credible intervals with borrowing are substantially narrower than those obtained without borrowing. This extra precision leads to increased statistical power and lower type 1 error when the observed treatment effects across regions are similar. Importantly, decreasing the range of the credible intervals by 10–20% corresponds to an increased effective trial sample size of 23–56%.

In example 2, the observed treatment effects differ by region, with data from the Region of the Americas and the European Region showing greater differences than in the first example. When the observed differences exceed random variation, the model attributes the observed differences to true underlying variation in the treatment effect across regions. Accordingly, the model borrows minimal information, both for moving the point estimates together and for shrinking the credible intervals, similar to the no borrowing model. In this example, the data from each region are more independently interpreted.

The behaviour of the hierarchical model is called dynamic borrowing, because the degree of borrowing is not prespecified, but depends on the similarity in the observed treatment effect across regions. Dynamic borrowing can have a significant impact on the statistical characteristics of a clinical trial. In this example, with 250 participants enrolled in each region, we would have only 70% power to detect a HR of 1.4 when separately analysing the regions, with the usual 2.5% one-sided type 1 error rate. Borrowing of information across regions both offers benefits and carries risks, depending on the underlying true treatment effects in each of the three regions.

Benefits from dynamic borrowing exist when the treatment works similarly across the regions, meaning the true HRs are approximately equal. For example, with our chosen level of borrowing, we obtain 90% power when all regions have the same HR of 1.4, considerably higher than the 70% power when separately analysing the regions. This benefit also applies to type 1 error. Should the HR be 1.0 (no effect) in all three regions, the type 1 error is reduced from 2.5% to 1.3%. This improvement in the statistical characteristics of the trial is a large inferential advantage compared to a trial that separately analyses the regions. The effects need not be equal to achieve such benefit. For example, assume when the experimental treatment is used in Africa the treatment effect is a HR of 1.4, while the HR in the Americas is 1.3 and in Europe 1.1. Even with lower hazard ratios in the other regions, the power of the trial to detect the treatment benefit in African participants is still increased from 70% to 79%. The increased precision of the estimates outweighs the potential bias from borrowing from data obtained in the other regions with slightly lower HRs. 

In contrast, if extremely large differences are expected in the true treatment effect across the three regions, using a hierarchical model has substantial risks. For example, if the HR in the African participants is 1.4 while the HR in both European and American participants is 1.0 (no effect), then the power of the analysis with borrowing for detecting the benefit in African participants is reduced from 70% to 55%. Similarly, if the HR in African participants is 1.0, while the HR in European and American participants is 1.4, the type 1 error in determining the treatment effect in Africa is increased to 11%. These risks are often of lesser concern, because it is unusual for a treatment to have no effect in one setting and a large effect in another. However, when choosing whether a hierarchical model is appropriate, one must assess whether small differences between countries are more likely than extreme differences. When small differences are expected, the benefits will exceed the risks, and when extreme differences are expected, the risks of using such an analysis may exceed the benefits.

## Challenges 

Efforts to increase the use of statistical approaches that yield valid estimates of treatment benefit for all participating populations are both scientifically and ethically important, however, these efforts face several challenges. Research funders and regulators may not consider the importance of addressing benefits across different populations or understand the strengths and limitations of the competing methodological approaches. In multicontinental studies that include low-resourced settings, regulators should consider requiring trial designs and analysis strategies that directly address the needs of the populations in the low-resourced settings, rather than allowing the needs of these populations to be treated as secondary considerations.

While generating sufficiently precise estimates of treatment effects across multiple regions require a larger sample size than a single estimate for all regions, a larger sample size accounts for the complexity of modern global clinical trials, ensuring that all participating populations can learn whether the treatment is effective for them. Modern inferential models, however, can be used to maximize the efficiency with which we can quantify differences in treatment effects across populations and settings, and thus avoid unnecessarily large sample sizes. Regulators, funders and others positioned to influence the standards for trials conducted in low-resource settings should strive to ensure the trials are well suited to provide information specific to those settings, helping to preserve justice for all participants.
